# Choice Requires Cognitive Availability: Protective Structure as an Enabling Condition for Autonomy Support in Digitally Distracted Physical Education Classrooms

**DOI:** 10.1002/pchj.70099

**Published:** 2026-05-26

**Authors:** Haiyan Xu, Bei Zhang, Wei Wei

**Affiliations:** ^1^ Mental Health Education and Counseling Center XiangXi Vocational and Technical College for Nationalities Xiangxi Tujia and Miao Autonomous Prefecture China; ^2^ School of Education Wuhan College of Arts and Sciences Wuhan China; ^3^ Faculty of Education Guangxi Normal University Guilin China

**Keywords:** autonomy support, cognitive availability, flow, identified regulation, physical education, protective structure

## Abstract

Does autonomy support retain its motivational benefits when digital distraction pervades the classroom? Drawing on self‐determination theory and cognitive‐resource perspectives, we proposed the Cognitive Availability Hypothesis: when attentional resources are depleted by smartphone presence, task choice cannot fulfill its motivational potential; protective structure—removing external digital distractions—functions as an enabling condition rather than a parallel instructional option. Using a fully counterbalanced 2 × 2 within‐subjects crossover design (*N* = 121 Chinese undergraduates; 8 weeks), we independently manipulated protective structure (smartphone sequestration: present vs. absent) and autonomy support (task choice: present vs. absent) in university badminton classes, with flow as the primary outcome and class engagement as a secondary outcome. The Sequestration × Choice interaction was significant for flow: task choice had a negligible effect without sequestration but a moderate effect with sequestration. A parallel pattern emerged for engagement. Process‐consistent analyses indicated that sequestration was associated with lower cognitive load and that the conditional choice effect on motivational regulation was most clearly expressed in identified regulation, the form of motivation that involves valuing and personally endorsing the activity. Manipulation checks confirmed that sequestration reduced perceived phone distraction without undermining perceived choice or choice authenticity. These findings suggest that in digitally saturated classrooms, establishing attentional conditions may precede, rather than merely complement, autonomy‐supportive instruction.

## 1 Introduction

In contemporary classrooms, a growing paradox has become difficult to ignore: many students do not lack the desire to learn, yet repeatedly struggle to sustain attention long enough to act on that desire. This pattern has been described as digital akrasia, in which students genuinely intend to remain focused but are continually pulled away by notifications, social media, and entertainment content (Aagaard 2020). At a deeper level, this pattern reflects recurring self‐regulatory failure: digital devices create persistent attentional competition, deplete executive resources, and thereby weaken students' capacity to resist further distraction, creating a self‐reinforcing cycle (Kostić and Ranđelović 2022). Consistent with this account, increasing smartphone dependence has been shown to impair university students' self‐regulated learning capacity (Gezgin et al. 2025). The challenge may be especially acute in physical education, where learning depends on the ongoing coupling of attention, perception, and movement; in such settings, attentional fragmentation disrupts not only information acquisition but the embodied learning process itself (Faella et al. 2025). For this reason, physical education instructors frequently rely on autonomy‐supportive strategies to re‐engage students, and research generally shows that autonomy support positively predicts autonomous motivation and attentional focus (Raabe et al. 2019). Yet an increasingly common field observation remains undertheorized: in digitally distracted classrooms, offering students choice does not always yield the motivational benefits that theory would lead us to expect.

Self‐Determination Theory (SDT; Deci and Ryan 2012) provides one of the most influential frameworks for understanding how teachers can support students' engagement. Within this framework, autonomy support emphasizes acknowledging students' perspectives, minimizing pressure, and providing meaningful opportunities for choice, with task choice representing one of its most direct operational forms (Patall and Zambrano 2019). Structure, in contrast, refers to the provision of clear expectations, guidance, and contingency information that help students navigate learning activities effectively. A substantial body of work has shown that both autonomy support and structure can independently promote engagement (Hospel and Galand 2016), and that their combination often produces the strongest outcomes across classroom contexts (Jang et al. 2010; Patall et al. 2008). Importantly, however, this literature was developed in contexts in which one premise could largely be taken for granted: students had sufficient attentional resources to perceive, process, and respond to autonomy‐supportive cues. In digitally saturated classrooms, this premise may no longer hold. When smartphones continuously compete for attention, the issue may not be whether autonomy support is theoretically beneficial, but whether students are cognitively available to benefit from it in the first place.

This possibility suggests that a form of structure not typically foregrounded in SDT research may become theoretically relevant in digitally distracted classrooms. Existing work has focused primarily on instructional structure—clarity, guidance, and predictable organization within the learning task itself (Jang et al. 2010). However, when personal devices function as ongoing attentional competitors, teachers may also need to protect the attentional conditions under which learning and motivation can occur. We refer to this as protective structure: an environmental arrangement designed to reduce external interference so that students can engage more fully with the task at hand. Conceptually, protective structure differs from controlling instruction. The former manages competition outside the task; the latter restricts how students participate within the task itself (Reeve and Jang 2006). This distinction is theoretically important because any attempt to reduce distraction by removing smartphones could be criticized as autonomy‐thwarting unless it can be shown to protect attention without undermining students' perceived choice.

On this basis, we propose the Cognitive Availability Hypothesis: in classrooms characterized by substantial digital distraction, the motivational benefits of task choice depend on whether attentional resources are sufficiently available. Choice is not motivationally cost‐free. To function as autonomy support, it must be noticed, evaluated, and experienced as personally meaningful rather than merely processed as an additional decision demand; these operations draw on limited executive resources (Baumeister 2002; Vohs et al. 2008), and when such resources are strained, choice may feel burdensome rather than autonomy‐supportive (Schwartz 2004). At the same time, smartphones deplete cognitive resources through multiple routes. The mere presence of one's smartphone reduces available cognitive capacity (Ward et al. 2017); students who use phones during class encode less information and perform more poorly on tests (Kuznekoff and Titsworth 2013); and effective phone‐management habits help explain the association between self‐control and academic performance (Troll et al. 2021). Conversely, removing phones can restore cognitive resources, with students in phone‐removed conditions outperforming those in other phone‐policy conditions (Lee et al. 2017). From the perspective of self‐regulated learning, this pattern is also theoretically coherent: learning depends on goal setting, metacognitive monitoring, and strategic adjustment, all of which require executive control resources (Pintrich 2000). When digital devices continually activate attentional competition, those resources may be insufficient not only for resisting distraction but also for processing autonomy‐supportive input in a motivationally meaningful way (Kostić and Ranđelović 2022). Together, these lines of evidence suggest a straightforward prediction: when phones remain present, the motivational effect of task choice should be attenuated; when attentional competition is reduced, the same choice should be more likely to promote deeper engagement and stronger internalization of the activity's value, particularly in the form of identified regulation (Vansteenkiste et al. 2018).

Physical education provides a particularly suitable context for an initial test of this hypothesis. Flow, the optimal experiential state characterized by deep absorption in an activity (Csíkszentmihályi 2002), is especially sensitive to attentional integrity; by definition, sustained distraction and flow are difficult to reconcile (Jackson et al. 2008). In embodied learning contexts such as badminton practice, the real‐time coupling of perception, decision making, and movement makes attentional resources functionally indispensable. For this reason, flow serves as a high‐sensitivity outcome for detecting whether autonomy support retains its motivational potential under varying attentional conditions. In the present study, we tested this hypothesis in a counterbalanced crossover study in university badminton classes, independently manipulating smartphone sequestration and task choice. Flow was specified as the primary outcome, with class engagement as a secondary outcome. The within‐subjects design was selected to control for stable individual differences, such as baseline self‐control and smartphone dependence, while increasing power to detect interaction effects. Based on the framework outlined above, we predicted a significant interaction between smartphone sequestration and task choice on flow and engagement (H1), such that the effect of task choice would be negligible or non‐significant when smartphones were not sequestered (H2a) but stronger when they were sequestered (H2b). We further expected that smartphone sequestration would be associated with lower cognitive load (H3), and that task choice would show a stronger positive association with identified regulation under smartphone sequestration than under smartphone presence (H4).

## 2 Method

### 2.1 Design and Analytic Unit

The study used a fully counterbalanced 2 × 2 within‐subjects crossover design in which protective structure (smartphone sequestration: present vs. absent) and autonomy support (task choice: present vs. absent) were independently manipulated. This produced four experimental conditions: control (CON), smartphone sequestration only (SS‐only), task choice only (TA‐only), and the combined condition (SS + TA). Each condition was implemented over two consecutive class meetings.

Because the manipulations were defined at the condition level and each condition extended across two class meetings, the two meeting‐level scores within each condition were averaged before the primary analyses. This yielded up to four phase‐level observations per participant, corresponding to the four crossover conditions. This approach aligned the analytic unit with the crossover design and reduced occasion‐specific noise from any single class meeting.

Condition order was counterbalanced across the four intact classes using a balanced Latin square, such that each condition appeared equally often in each period position. No washout period was introduced because the manipulations were immediate classroom arrangements with no plausible residual effect beyond the class session; instead, period was modeled statistically in the primary analyses. The instructor received procedural instructions before each phase but was not informed of the study hypotheses.

### 2.2 Participants and Setting

Participants were undergraduate students enrolled in an elective badminton course at a large public university in southern China. All four classes were taught by the same instructor in the same indoor facility, which minimized instructor‐ and setting‐related variation.

A total of 128 students provided informed consent. Following the phase‐based analytic plan, students who missed two or more experimental phases were excluded from the primary analyses, resulting in a final analytic sample of 121 students (40.5% men; M_age_ = 19.23 years, SD = 0.88). A sensitivity analysis retaining all consented students with available data was prespecified. An a priori power analysis conducted in G*Power 3.1 indicated that 82 participants would provide 90% power to detect a medium within‐subjects interaction effect (*f* = 0.25) at *α* = 0.05, assuming a within‐person correlation of 0.50. The final sample exceeded this target. The study was approved by the institutional ethics committee, and all participants provided written informed consent before participation.

### 2.3 Experimental Manipulations and Fidelity

#### 2.3.1 Smartphone Sequestration (Protective Structure)

In sequestration phases, a teaching assistant collected students' smartphones before class and stored them in a visible numbered pouch at the front of the gym. Phones were returned only after class and after questionnaire completion. To minimize the possibility that sequestration would be experienced as controlling, the instructor introduced this procedure using autonomy‐supportive wording (e.g., emphasizing immersion and freedom from interruption) rather than compliance‐based or disciplinary language (Reeve and Jang 2006). In non‐sequestration phases, smartphones were not mentioned and students retained them as usual.

#### 2.3.2 Task Choice (Autonomy Support)

In task‐choice phases, before each technical drill the instructor offered two or three practice options that were equivalent in instructional goal and duration but differed in format or situational emphasis (e.g., straight vs. diagonal footwork patterns). Options were presented as equally legitimate, and the number of options was intentionally limited to avoid excessive decisional burden (Patall et al. 2008). In no‐choice phases, the instructor assigned a single drill that was always one of the drills used in the corresponding choice phase, thereby maintaining instructional content equivalence while removing the choice element.

#### 2.3.3 Fidelity

Implementation fidelity was monitored through structured classroom observations conducted by a research assistant who was not involved in data analysis. Eight unannounced observations were completed across the experimental period, covering all four conditions. Observed sessions met the predefined fidelity criteria for the presence or absence of smartphone sequestration and task choice.

### 2.4 Measures

Unless otherwise noted, all measures were administered as state reports referring to the current class session, with higher scores indicating higher levels of the construct. Internal consistency estimates (Cronbach's α) reported below were computed at the phase level and pooled across the four experimental conditions in the present sample.

#### 2.4.1 Flow State

Flow state, the primary outcome, was measured with the 9‐item Short Flow State Scale (S‐FSS‐2; Jackson et al. 2008), administered in its Chinese version (Liu 2010). The S‐FSS‐2 includes one item for each of the nine flow dimensions (e.g., challenge–skill balance, action–awareness merging, autotelic experience), and items refer to participants' experience during the current class. Responses were rated on a 5‐point scale (1 = *strongly disagree*, 5 = *strongly agree*). Internal consistency was acceptable in the present sample (*α* = 0.85).

#### 2.4.2 Class Engagement

Class engagement, the secondary outcome, was assessed with three state items developed for the present study to capture behavioral participation during the current class (e.g., “I participated actively in today's practice”). Item development was guided by Skinner et al.'s (2009) conceptualization of behavioral engagement and by indicators in the Student Course Engagement Questionnaire (Handelsman et al. 2005). Items were rated on a 5‐point scale (1 = *strongly disagree*, 5 = *strongly agree*). Because the crossover design required sensitivity to within‐person variation across phases, a brief state‐focused format was used instead of semester‐level course‐engagement instruments. Internal consistency was good (*α* = 0.82).

#### 2.4.3 Motivation Regulation

Situational motivation was assessed with the 16‐item Situational Motivation Scale (SIMS; Guay et al. 2000), comprising four 4‐item subscales: intrinsic motivation, identified regulation, external regulation, and amotivation. To maintain a common response format across measures and to reduce response burden in the end‐of‐class assessment, items were rated on a 5‐point scale rather than the original 7‐point scale. Internal consistency in the present sample was acceptable to good across subscales: intrinsic motivation (*α* = 0.86), identified regulation (*α* = 0.81), external regulation (*α* = 0.78), and amotivation (*α* = 0.83). Following recent recommendations against collapsing qualitatively distinct motivational regulations into a single composite score (Chemolli and Gagné 2014; Howard et al. 2020), the four subscales were analyzed separately rather than combined into a Relative Autonomy Index. Identified regulation was specified a priori as the focal motivational process variable because it most directly reflects valuing and personally endorsing the activity (Vansteenkiste et al. 2018).

#### 2.4.4 Cognitive Load

Cognitive load was assessed with the single‐item 9‐point mental‐effort rating (Paas 1992; Paas et al. 1994), which has been widely used as a state measure of cognitive load in instructional research and shows acceptable test–retest reliability and convergent validity with multi‐item indices (Van Gog and Paas 2008). The item was preceded by a brief instruction distinguishing mental effort from physical exertion and ranged from 1 (*very, very low mental effort*) to 9 (*very, very high mental effort*). Because this measure consists of a single item, internal consistency is not reported.

#### 2.4.5 Manipulation Checks

Three single‐item manipulation checks were administered. Perceived phone distraction assessed the extent to which the participant felt distracted by their phone during the class. Perceived choice assessed how much choice the participant experienced during practice. Perceived authenticity of choice assessed whether the perceived choice felt genuine; this item was administered only in task‐choice phases. The perceived choice and choice authenticity items were informed by the autonomy‐supportive vs. controlling distinction described by Reeve and Jang (2006). All items were rated on a 7‐point scale. Perceived choice was measured across all four conditions because a central theoretical question was whether smartphone sequestration changed the attentional environment without reducing students' experienced autonomy.

### 2.5 Procedure

The study was conducted over 9 weeks during the spring semester. Week 1 was used for recruitment, consent, and demographic data collection. Weeks 2–9 comprised the four experimental phases.

Each class session followed the same basic structure: warm‐up (10 min), technical practice (25 min), cool‐down (5 min), and questionnaire completion (approximately 5 min). The task‐choice manipulation, when present, was implemented during the technical‐practice segment.

Because smartphones were unavailable during sequestration phases, questionnaires in those phases were completed using paper‐and‐pencil forms. In non‐sequestration phases, questionnaires were completed electronically via QR code. This mode difference was an unavoidable design constraint created by the manipulation itself. To address this issue, robustness analyses were planned using within‐person contrasts that were internally mode‐consistent.

Students were informed that the study concerned how routine instructional arrangements related to in‐class experiences. The specific hypotheses regarding cognitive availability and enabling conditions were not disclosed.

### 2.6 Analytic Strategy

Primary analyses were conducted on phase‐level data, with the four crossover phases nested within participants. Models were estimated in R using linear mixed‐effects modeling with lme4, and inferential tests with denominator degrees of freedom were obtained via lmerTest using the Satterthwaite approximation.

For the primary outcomes (flow and class engagement), each model included fixed effects of smartphone sequestration (SS), task choice (TA), their interaction (SS × TA), period, and Latin‐square sequence, with participant entered as a random intercept. The key test of the central hypothesis was the SS × TA interaction. When interactions were significant, simple effects of task choice were examined separately at each level of smartphone sequestration using estimated marginal means and 95% confidence intervals.

Manipulation checks were analyzed using the same modeling structure. For the process‐consistent analyses, cognitive load and each SIMS subscale were modeled in separate mixed‐effects models using the same fixed‐ and random‐effects structure. Identified regulation was treated as the primary motivational process variable, given its theoretical centrality to the present hypothesis. To examine whether the observed process variables covaried with the primary outcome in the predicted direction, additional models entered cognitive load or identified regulation as concurrent time‐varying covariates predicting flow.

To maximize interpretability, the primary results were reported as unstandardized model estimates, estimated marginal means, and 95% confidence intervals. Standardized paired effect sizes for simple contrasts were provided as descriptive supplements rather than as the primary basis for inference. Because response mode was aligned with the sequestration manipulation, robustness to mode confounding was evaluated using a within‐person difference‐in‐differences contrast comparing the effect of task choice under sequestration versus non‐sequestration conditions.

## 3 Results

### 3.1 Preliminary Analyses and Descriptive Statistics

The final sample contributed 476 phase‐level observations (98.3% of 484 possible). Null‐model ICCs indicated meaningful between‐person variation for flow (0.40) and engagement (0.35), justifying the multilevel approach. Descriptive statistics are presented in Table 1. Flow and engagement were highest in the SS + TA condition and lowest in CON, while cognitive load showed the reverse pattern across the smartphone manipulation. Inter‐subscale correlations among the SIMS dimensions followed the expected SDT simplex (intrinsic–identified *r* = 0.49; identified–external *r* = 0.18; intrinsic–amotivation *r* = −0.34), supporting construct validity. Estimated marginal means from the models below differed slightly from raw cell means (typically 0.01–0.04 units), reflecting adjustment for period and sequence.

**TABLE 1:** Descriptive statistics across experimental conditions (*N* = 121).

Variable	Range	CON	SS‐only	TA‐only	SS + TA
Flow	1–5	3.28 (0.80)	3.50 (0.78)	3.42 (0.82)	3.86 (0.76)
Class engagement	1–5	3.32 (0.78)	3.46 (0.71)	3.43 (0.82)	3.78 (0.69)
Intrinsic motivation	1–5	3.45 (0.83)	3.55 (0.74)	3.58 (0.79)	3.74 (0.71)
Identified regulation	1–5	3.20 (0.81)	3.35 (0.70)	3.32 (0.79)	3.70 (0.68)
External regulation	1–5	2.62 (0.85)	2.58 (0.79)	2.51 (0.84)	2.45 (0.77)
Amotivation	1–5	2.08 (0.85)	1.92 (0.78)	2.05 (0.82)	1.88 (0.74)
Cognitive load	1–9	4.72 (1.68)	3.48 (1.71)	4.58 (1.74)	3.61 (1.65)
Perceived distraction	1–7	3.71 (1.52)	2.34 (1.41)	3.58 (1.49)	2.29 (1.38)
Perceived choice	1–7	2.18 (1.14)	2.31 (1.09)	5.41 (1.22)	5.63 (1.18)

*Note:* Values are M (SD). Identified regulation was specified a priori as the focal motivational process variable.

Abbreviations: CON, control; SS + TA, combined; SS‐only, smartphone sequestration only; TA‐only, task choice only.

### 3.2 Manipulation Checks

Manipulation checks indicated that the experimental manipulations operated as intended. Smartphone sequestration significantly reduced perceived phone distraction, M = 2.32 versus 3.65, *F*(1, 354) = 54.23, *p* < 0.001, *η*_p_^2^ = 0.133, with no significant task‐choice main effect or SS × TA interaction, *ps* > 0.50. Task choice significantly increased perceived choice, M = 5.52 versus 2.25, *F*(1, 354) = 402.43, *p* < 0.001, *η*_p_^2^ = 0.532. Smartphone sequestration did not significantly affect perceived choice, *b* = 0.13, SE = 0.12, *p* = 0.306, nor did it interact with task choice, *p* = 0.614. Within task‐choice phases, 88.6% of participants rated the available choices as genuine, and choice authenticity did not differ significantly by sequestration condition, *F*(1, 119) = 1.14, *p* = 0.288. These results suggest that smartphone sequestration reduced phone‐related distraction while leaving students' perceived choice and choice authenticity statistically unchanged.

### 3.3 Interaction of Sequestration and Task Choice on Flow (H1, H2)

The primary hypothesis was tested with a linear mixed‐effects model including SS, TA, their interaction, period, and sequence as fixed effects, and participant as a random intercept. Consistent with H1, the SS × TA interaction was significant, *b* = 0.22, SE = 0.10, 95% CI [0.03, 0.41], *F*(1, 354) = 4.84, *p* = 0.028, *η*_p_^2^ = 0.014. The SS main effect was also significant, *b* = 0.22, SE = 0.08, *F*(1, 354) = 7.56, *p* = 0.006, *η*_p_^2^ = 0.021, whereas the TA main effect was not, *b* = 0.14, SE = 0.08, *F*(1, 354) = 3.06, *p* = 0.081, *η*_p_^2^ = 0.009. Marginal and conditional *R* ^2^ were 0.07 and 0.47. Full results appear in Table 2.

**TABLE 2:** Linear mixed‐effects model results for flow state.

Effect	*b*	SE	95% CI	*F* (1, 354)	*p*	*η*_p_^2^ [90% CI]
Intercept (CON)	3.28	0.08	[3.13, 3.43]	—	—	—
SS	0.22	0.08	[0.06, 0.38]	7.56	0.006	0.021 [0.003, 0.053]
TA	0.14	0.08	[−0.02, 0.30]	3.06	0.081	0.009 [0.000, 0.031]
SS × TA	0.22	0.10	[0.03, 0.41]	4.84	0.028	0.014 [0.000, 0.041]

*Note:* Period and sequence were included as fixed effects but are omitted for clarity. *τ* ^2^ = 0.28; *σ* ^2^ = 0.34. Marginal *R* ^2^ = 0.07; conditional *R* ^2^ = 0.47.

**FIGURE 1:**
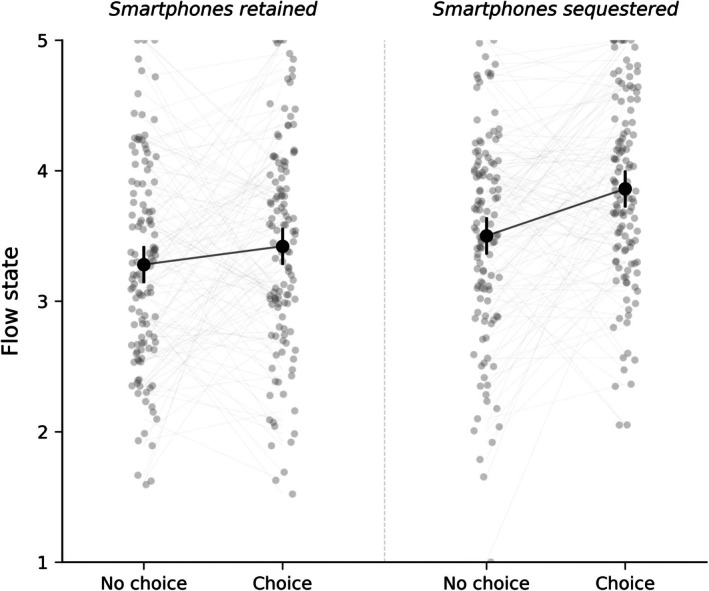
Flow state across experimental conditions: individual observations and estimated marginal means. Hollow points represent individual phase‐level observations (jittered horizontally). Dashed lines connect within‐subject paired values under each smartphone condition. Black points indicate estimated marginal means; error bars represent 95% confidence intervals. Connecting lines between means illustrate the task‐choice effect within each condition.

Decomposing the interaction (H2; Bonferroni‐adjusted *α* = 0.025), simple effects revealed an asymmetric pattern. Without sequestration, task choice did not predict flow, *b* = 0.14, SE = 0.08, *t*(354) = 1.75, *p* = 0.081, *d* = 0.18. With sequestration, the identical manipulation produced a significant effect, *b* = 0.36, = 0.08, *t*(354) = 4.50, *p* < 0.001, *d* = 0.46. Figure 1 displays individual observations and estimated marginal means across the four conditions. This pattern is informative beyond the size of the interaction.

### 3.4 Parallel Pattern on Class Engagement

A convergent pattern emerged for engagement. The SS × TA interaction was significant, *b* = 0.21, SE = 0.09, *F*(1, 354) = 5.44, *p* = 0.020, *η*_p_^2^ = 0.015. Simple effects mirrored those for flow: task choice did not predict engagement without sequestration, *b* = 0.11, *t*(354) = 1.38, *p* = 0.169, *d* = 0.13, but produced a significant positive effect with sequestration, *b* = 0.32, *t*(354) = 4.57, *p* < 0.001, *d* = 0.42. Replication across two outcomes provides convergent support for the central hypothesis and reduces concern that the interaction is instrument‐specific.

### 3.5 Cognitive Load as a Process Variable (H3)

Consistent with H3, sequestration substantially lowered cognitive load (sequestration M = 3.55, no sequestration M = 4.65), *b* = −1.10, SE = 0.20, *F*(1, 354) = 30.25, *p* < 0.001, *η*_p_^2^ = 0.079. Neither the TA main effect (*F* = 0.07, *p* = 0.788) nor the interaction (*F* = 1.29, *p* = 0.257) was significant, indicating the reduction was specific to the smartphone manipulation.

Entering cognitive load as a concurrent time‐varying covariate in the flow model, it negatively predicted flow, *b* = −0.08, SE = 0.03, *p* = 0.008, and the SS main effect attenuated from *b* = 0.22 to *b* = 0.15 (31.8% reduction), while remaining significant. The implied indirect estimate (*a* × *b* = 0.088) closely matched this attenuation. Because cognitive load and flow were measured at the same time point, we interpret this as consistent with a cognitive pathway.

### 3.6 Identified Regulation as a Focal Motivational Process (H4)

Turning to motivational regulation (H4), the SS × TA interaction was significant for identified regulation, *b* = 0.23, SE = 0.10, *F*(1, 354) = 5.29, *p* = 0.022, *η*_p_^2^ = 0.015. Simple effects paralleled those for flow: task choice did not predict identified regulation without sequestration, *b* = 0.12, *t*(354) = 1.71, *p* = 0.088, *d* = 0.16, but produced a significant increase with sequestration, *b* = 0.35, *t*(354) = 5.00, *p* < 0.001, *d* = 0.49.

Parallel analyses on the remaining SIMS subscales revealed no significant interactions for intrinsic motivation (*F* = 0.36, *p* = 0.549), external regulation (*F* = 0.18, *p* = 0.672), or amotivation (*F* = 0.05, *p* = 0.823). Two main effects were nevertheless detectable: task choice was associated with a small reduction in external regulation (*b* = −0.12, *p* = 0.085), and sequestration with lower amotivation (*b* = −0.16, *p* = 0.021). Neither, however, displayed the SS × TA interaction observed for identified regulation, supporting the interpretation that the conditional choice effect is most clearly expressed in the regulation type involving valuing and personally endorsing the activity. When identified regulation was entered as a concurrent covariate in the flow model, it positively predicted flow, *b* = 0.30, SE = 0.07, *p* < 0.001, and the SS × TA coefficient for flow attenuated from 0.22 to 0.16 (27.3% reduction).

### 3.7 Robustness Analyses

Three robustness analyses evaluated the stability of the core interaction. First, retaining period and sequence as fixed effects left the SS × TA interaction on flow significant, *F*(1, 350) = 4.68, *p* = 0.031, *η*_p_^2^ = 0.013, with simple effects substantively unchanged. Period showed a marginal trend (*p* = 0.051), suggesting modest fatigue or practice effects, whereas sequence was not significant, *F*(3, 350) = 1.42, *p* = 0.236, with an unsystematic pattern across positions. Second, entering survey mode (paper vs. electronic) as a covariate yielded a non‐significant mode effect, *b* = 0.07, SE = 0.08, *p* = 0.402, with all core inferences retained. Finally, a within‐person difference‐in‐differences contrast—internally mode‐consistent by construction—computed task‐choice effects separately under each SS condition: ΔnoSS = Flow(TA‐only) − Flow(CON), Δ_SS_ = Flow(SS + TA) − Flow(SS‐only). The contrast ΔΔ = Δss − Δ_noSS_ was significant for flow, ΔΔ = 0.22, *t*(120) = 3.10, *p* = 0.002, *d* = 0.28, and engagement, ΔΔ = 0.21, *t*(120) = 2.78, *p* = 0.006, *d* = 0.25. This mode‐consistent contrast further ruled out response mode as the explanation for the interaction.

## 4 Discussion

The central finding can be characterized as a conditional replication. Task choice produced the motivational benefits documented in prior meta‐analytic work (Patall et al. 2008), but only when smartphones were sequestered. Without sequestration, the identical manipulation was effectively inert (*d* = 0.18, *p* = 0.081); with sequestration, it produced a moderate effect (*d* = 0.46, *p* < 0.001), and this asymmetry was paralleled in class engagement. The effect of choice did not merely attenuate in the presence of phones; it shifted from non‐significant to significant across the two attentional conditions. Such a qualitative shift is more consistent with an enabling‐condition account than with a graded‐moderation account, under which a reduced but still detectable choice effect would be expected without sequestration.

### 4.1 Autonomy Support and Structure: An Enabling Condition Within the Synergy Model

The present data are broadly consistent with Jang et al.'s (2010) synergy model: the combined SS + TA condition produced the highest flow and engagement. However, the data also reveal a pattern that the synergy model does not explicitly predict. Under an additive or simply‐synergistic account, choice would produce some detectable effect even without sequestration. The observed on–off asymmetry departs from this expectation.

One interpretation is that the synergy documented in earlier classroom research may have tacitly depended on a condition that those contexts naturally satisfied—namely, that students possessed sufficient attentional resources to process autonomy‐supportive cues. In digitally saturated classrooms, this condition may no longer hold by default. Framed this way, the present findings do not challenge the synergy model but specify a boundary condition under which its predictions may be attenuated. The relationship between structure and autonomy support in digitally distracted settings may be hierarchical rather than symmetric. This reframing is compatible with the collaborative perspective articulated by Hospel and Galand (2016), in which each contributes to engagement while allowing their relative contribution to vary with the cognitive demands of the learning environment.

### 4.2 A Cognitive Pathway Consistent With Brain‐Drain Findings

The cognitive‐load pathway is consistent with Ward et al.'s (2017) demonstration that smartphone presence reduces available cognitive capacity. The present study extends this effect in two ways: from laboratory cognitive tasks to authentic classroom learning, and from cognitive performance to the motivational responsiveness to autonomy‐supportive cues. Imposing cognitive load during motor‐skill acquisition has been shown to eliminate the benefits of self‐controlled practice (Couvillion et al. 2020); the present findings suggest an analogous dynamic for motivational interventions.

It should be acknowledged that the magnitude of brain‐drain effects has not always been recovered in subsequent work, with some heterogeneity across studies. The present findings speak to this debate indirectly: rather than replicating the cognitive‐performance effect itself, they document downstream motivational consequences that would be expected only if some form of attentional competition were operating.

### 4.3 Identified Regulation as the Focal Motivational Process

The motivational‐regulation results offer a finer‐grained picture. Among the four regulations, the conditional pattern was most clearly expressed in identified regulation, while interactions on intrinsic motivation, external regulation, and amotivation did not reach significance. This specificity should be interpreted cautiously: differences between significant and non‐significant results are not themselves necessarily significant (Gelman and Stern 2006), and formal tests of dissociation would require larger samples.

Nonetheless, the pattern is theoretically coherent. Identified regulation requires learners to appraise an activity's personal relevance and integrate it with their values—operations that draw on working memory and reflective cognitive processing. Intrinsic motivation, by contrast, can be sustained by relatively automatic affective responses to the activity itself, placing lower demands on cognitive resources. If smartphone presence primarily depletes resources required for effortful cognitive appraisal rather than those supporting automatic affective engagement, the regulations most dependent on such appraisal—identified regulation among them—would be expected to show the greatest sensitivity to the attentional environment (Vansteenkiste et al. 2018). The present data are consistent with this prediction, though direct testing awaits designs that can separate the temporal dynamics of cognitive restoration and motivational shift within a single session.

### 4.4 Situating the Findings Among Prior Work on Smartphones in PE

The present findings appear to stand in tension with prior work suggesting that smartphones can positively support physical education learning (Buchner and Zumbach 2018; Koekoek and Van Hilvoorde 2018). These findings are not in conflict with the present data so much as they illuminate a critical distinction. In both cited studies, the smartphone was the instructional medium—attention was directed toward the device as a learning tool. In the present study, smartphones were present as social and entertainment platforms competing with the instructional task.

The critical variable is thus not smartphone presence per se but whether the device functions as an attentional ally or competitor. This distinction maps onto the classical differentiation between germane and extraneous cognitive load (Sweller 1988): task‐directed smartphone use may contribute germane load that supports learning, whereas off‐task smartphone availability constitutes extraneous load that depletes resources required for autonomous engagement. Protective structure is therefore expected to be beneficial when phones operate as distractors but potentially counterproductive when phones operate as instructional tools—a prediction consistent with both literatures and one the present framework generates for future testing.

### 4.5 Protective Structure Is Not Controlling Instruction

A theoretically critical question is whether sequestration functioned as a controlling intervention that undermined the autonomy it was meant to enable. The manipulation‐check data speak directly to this concern. Sequestration reduced perceived phone distraction (*η*_p_^2^ = 0.13) while having no detectable effect on perceived choice (*p* = 0.306) or choice authenticity (88.6% rated choices as genuine regardless of condition). This null effect is substantively meaningful. It suggests that the distinction between protective structure—managing attentional competition external to the task—and controlling instruction—restricting how students participate within the task (Reeve and Jang 2006)—is operationally achievable, provided the intervention is framed in autonomy‐supportive rather than disciplinary language. Whether this boundary holds across cultural contexts with different norms around institutional authority remains an open question, particularly given that the high acceptance observed here may partly reflect educational norms specific to the Chinese context.

### 4.6 Practical Implications

The practical implication is specific rather than general. An effect size of *d* = 0.18 for task choice without sequestration is unlikely to justify the instructional investment required to design meaningful choice architectures, and would be difficult to detect reliably in typical classroom samples. The same intervention yielded *d* = 0.46 once phones were removed. This asymmetry supports what might be termed an attentional readiness principle: before activating motivational strategies, educators should consider whether the cognitive conditions that allow those strategies to operate are in place. Under this framing, phone management is reconceptualized not as a disciplinary measure but as motivational infrastructure—a precondition for effective teaching rather than a supplement to it.

### 4.7 Limitations and Future Directions

Several limitations qualify these conclusions. First, mediators and outcomes were assessed concurrently; the proposed sequential account—cognitive restoration enabling motivational internalization—requires within‐session experience sampling for direct testing. Second, survey mode and sequestration were partially confounded (paper in SS‐present phases, electronic in SS‐absent phases); although sensitivity analyses and a mode‐consistent difference‐in‐differences contrast supported the core interaction, future replications should administer measures via a mode independent of the manipulation. Third, the sample comprised Chinese undergraduates in a single PE course with a single instructor; generalization to lecture‐based courses, younger learners, and cultures where sequestration may be perceived as more autonomy‐threatening requires direct investigation. Fourth, only one form of autonomy support (task choice) and one form of protective structure (phone collection) were manipulated; dose–response examinations (e.g., phone muted vs. in bag vs. stored outside) would clarify whether the enabling condition operates as a threshold or a continuum.

## 5 Conclusion

Autonomy‐supportive instruction does not operate in an attentional vacuum. The present findings suggest that in digitally saturated classrooms, the cognitive prerequisites for task choice to function as a motivational cue may be absent by default, and that protective structure can restore these prerequisites without compromising students' experienced autonomy. For researchers, this points toward cognitive availability as a theoretically tractable boundary condition for autonomy support effects; for practitioners, it suggests that the sequencing of environmental protection and motivational strategy may matter as much as the strategies themselves.

## Funding

This work was supported by the Hunan Provincial Research Project on Ideological and Political Education in Higher Education [Grant No. 25D90], the Education Science Planning Project of Hunan Province (14th Five‐Year Plan) [Grant No. XJK25BZY067], and the Scientific Research Project of the Hunan Provincial Department of Education [Grant No. 24C1240].

## Disclosure

No potential conflict of interest was reported by the authors. This work was supported by Grant Nos. XJK25BZY067 and 24C1240.

## Ethics Statement

This study was conducted in accordance with the ethical principles of the Declaration of Helsinki and was approved by the Institutional Review Board of XiangXi Vocational And Technical College For Nationalities.

## Consent

All participants provided informed consent for the publication of their anonymized data and results in academic journals and related academic communications.

## Conflicts of Interest

The authors declare no conflicts of interest.

## Data Availability

The dataset is not publicly available as the informed consent obtained from participants did not include authorization for open data sharing. Researchers seeking access to de‐identified data summaries for academic purposes may contact the corresponding author, subject to institutional approval.
